# Recovery of the Structure and Function of the Pig Manure Bacterial Community after Enrofloxacin Exposure

**DOI:** 10.1128/spectrum.02004-21

**Published:** 2022-05-23

**Authors:** Tao Chen, Gaomiao Xie, Jiandui Mi, Xin Wen, Zhen Cao, Baohua Ma, Yongde Zou, Na Zhang, Yan Wang, Xindi Liao, Yinbao Wu

**Affiliations:** a National Engineering Research Center for Breeding Swine Industry, College of Animal Science, South China Agricultural University, Guangzhou, China; b Guangdong Laboratory for Lingnan Modern Agriculture, Guangzhou, China; c Maoming Branch, Guangdong Laboratory for Lingnan Modern Agriculture, Maoming, Guangdong, China; d Guangdong Provincial Key Lab of Agro-Animal Genomics and Molecular Breeding, Guangzhou, Guangdong, China; e Guangdong Engineering Technology Research Center of Harmless Treatment and Resource Utilization of Livestock Waste, Yunfu, Xinxing, China; f Foshan Customs Comprehensive Technology Center, Foshan, Guangdong, China; g Wen’s Foodstuff Group Co., Ltd., Yunfu, Xinxing, China; University of Mississippi

**Keywords:** enrofloxacin, functional gene, manure bacterial community, metagenomic sequencing, pig

## Abstract

At present, growth-promoting antibiotics are banned in the pig industry in many countries, but therapeutic antibiotics can still be used normally. However, the effect of therapeutic antibiotics on the structure and function of the intestinal bacterial community and its recovery is still unclear. We analyzed the effects of enrofloxacin on the pig manure bacterial community and functional genes during dosing and without dosing. Enrofloxacin caused significant changes in community structure. The changes in the diversity and structure of the bacterial community were the most obvious on the fifth day, and most of the differentially abundant genera (19/29) belonged to Firmicutes. The structure of the manure bacterial community in the low concentration enrofloxacin group was completely reverted after 10 days of drug discontinuation. In addition, enrofloxacin had a significant impact on the abundance of bacterial functional genes. Most of the differentially abundant functional genes of the manure bacterial community were significantly enriched, especially genes related to metabolic pathways, for adaptation to the antibiotic environment. Moreover, exposure to enrofloxacin increased the abundance of functional genes related to nitrogen metabolism in the manure bacterial community, and the total nitrogen content of pig manure was significantly reduced. The functional genetic differences caused by enrofloxacin exposure were completely reverted 10 days after drug discontinuation. The results of the present study suggest that enrofloxacin induces changes in the structure and function of manure bacterial communities, which may be rapidly recovered after drug discontinuation.

**IMPORTANCE** A stable intestinal bacterial community balance is beneficial for animal health. Enrofloxacin is widely used in animal husbandry as a therapeutic drug, but it can cause intestinal environmental imbalance. Enrofloxacin is widely present in groundwater, pork, etc., which leads to a greater risk of human exposure. The effect of enrofloxacin on the structure and function of the intestinal bacterial community and its recovery is still unclear. In this study, we found that enrofloxacin, as a therapeutic drug, can enhance nitrogen metabolism in the manure bacterial community. Moreover, the structure and function of the manure bacterial community in the low concentration enrofloxacin group may be completely reverted 10 days after drug discontinuation. This study provides a reference for the effect of enrofloxacin exposure on the intestinal bacterial community.

## INTRODUCTION

The intestinal bacterial community is a complex and balanced ecosystem (1). The complex combination of bacteria in the intestinal tract plays a key role in controlling health and disease in the host. The intestinal bacterial community has the functions of preventing pathogen growth, protecting the host, improving intestinal health, and regulating the physiological functions of the host (2). In addition, the role of the intestinal bacterial community in basic growth functions such as digestion, absorption, health, and other physiological functions is well known (3). Perturbations in the intestinal bacterial community can lead to host infection (4), inflammation, neurological disorders (5), and a decline in production performance (6). An increasing number of studies have found that antibiotics, nutrition, and other factors affect the composition of the intestinal bacterial community (7).

Antibiotics are currently the most effective treatment for diseases and are widely used in humans and livestock. At present, growth-promoting antibiotics are banned in animal husbandry in many countries, but therapeutic antibiotics can still be used normally (8, 9). Antibiotics can improve the health of the host, but they can also affect commensal bacteria while killing the target pathogen (10). Antibiotics may disturb the balance of the intestinal bacterial community. For example, the diversity of the intestinal bacterial community is reduced, and the community structure undergoes major changes, after lincomycin treatment (11). Similar observations were reported with ceftiofur (12), amoxicillin (13), etc. At present, increasing attention is being given to the effects of antibiotic stress on the intestinal bacterial community. However, most studies are still limited to the effect of antibiotics on the structure of the intestinal bacterial community during treatment, and few studies have focused on whether an antibiotic treatment has a sustained effect on the intestinal bacterial community (14). In addition, the influence of antibiotics on the intestinal bacterial community is greatly affected by the type of antibiotics, and the duration of treatment is different for different antibiotics. Some antibiotics have only a short-term effect on the intestinal bacterial community, while others may have a long-term effect (12, 13). In addition, the intestinal bacterial community represents a complex ecosystem performing essential functions in its host. Most of these functions are interconnected and closely intertwined with the host's physiology. For example, short-chain fatty acids, the product of microbial fermentation, are important substrates of intestinal cells and play an important role in the process of immune regulation (15). Recent studies have shown that antibiotics affect the abundance of bacterial functional genes, thereby affecting bacterial functions (16). However, a comprehensive understanding of the changes in the function of the intestinal bacterial community under antibiotic exposure is lacking.

Enrofloxacin, a second-generation fluoroquinolone, is widely used in animal husbandry as a therapeutic antibiotic (17). In 2013, the use of enrofloxacin in Chinese pig farms was as high as 3,090 tons (18). It has a good pharmacokinetic profile and excellent activity against Gram-negative aerobic bacteria and some Gram-positive bacteria (19) and can be used to treat piglet yellow-white dysentery, mycoplasma pneumonia, pleuropneumonia, salmonellosis, and various systemic infections caused by Escherichia coli (20, 21). However, due to the use of antibiotics, antibiotic resistance is becoming more and more serious, and enrofloxacin is no exception. This leads to the phenomenon that the actual clinical dosage of enrofloxacin is relatively high (22, 23). The use of enrofloxacin as a therapeutic drug can lead to the accumulation of residual enrofloxacin in pork. Blesa et al. (24) detected enrofloxacin residue at 42 μg/kg in Portuguese pork samples. Moreover, enrofloxacin is also one of the most common antibiotics detected in the aquatic environment in China. The detection rate of enrofloxacin in groundwater is as high as 67/87, and the concentration range is 2.7 to 49 ng/L (25, 26). Enrofloxacin may enter the human intestines through the consumption of pork and drinking water (27). Therefore, enrofloxacin was selected as an antimicrobial of interest for this study. A therapeutic dose of enrofloxacin is capable of significantly reducing the intestinal E. coli wild-type population (10). In addition, similar findings have been reported in *in vitro* studies, in which enrofloxacin significantly reduced the number of sensitive E. coli populations, which were replaced by E. coli strains with increased MIC values of enrofloxacin (28). At present, research on the effect of enrofloxacin on the intestinal bacterial community is incomplete.

Therefore, the purpose of this study was to investigate the effect of enrofloxacin on the intestinal bacterial community. The concentrations of enrofloxacin in the jejunum (1.7 ± 0.3 μg/mL), ileum (1.6 ± 0.7 μg/mL), cecum (2.1 ± 0.1 μg/mL), and colon (1.7 ± 0.1 μg/mL) of pigs were not as different after injection of 2.5 kg/mg body weight (23). The bacterial communities of pig manure and large intestine are highly similar. (29). The manure bacterial community is often used to represent the intestinal bacterial community. Many previous studies have directly referred to the manure bacterial community as the intestinal bacterial community or analyzed the intestinal bacterial community by examining manure (30–34). Therefore, in this study, fresh pig manure samples were collected before, during, and after enrofloxacin treatment, and the changes in bacterial community structure and function were analyzed through 16S rRNA and metagenome sequencing. This study systematically evaluated the toxicological effects of enrofloxacin on the pig manure bacterial community and provides a reference for the scientific and rational use of antibiotics in animal husbandry. Not only are humans and pigs similar in physiological attributes but the intestinal bacterial communities of humans and pigs are more similar than those of humans and mice (35, 36). Hence, this study also provided a reference for the destructive effect of enrofloxacin on the human intestinal bacterial community.

## RESULTS

### Influence of antibiotic stress on the pig manure bacterial community diversity.

**FIG 1:**
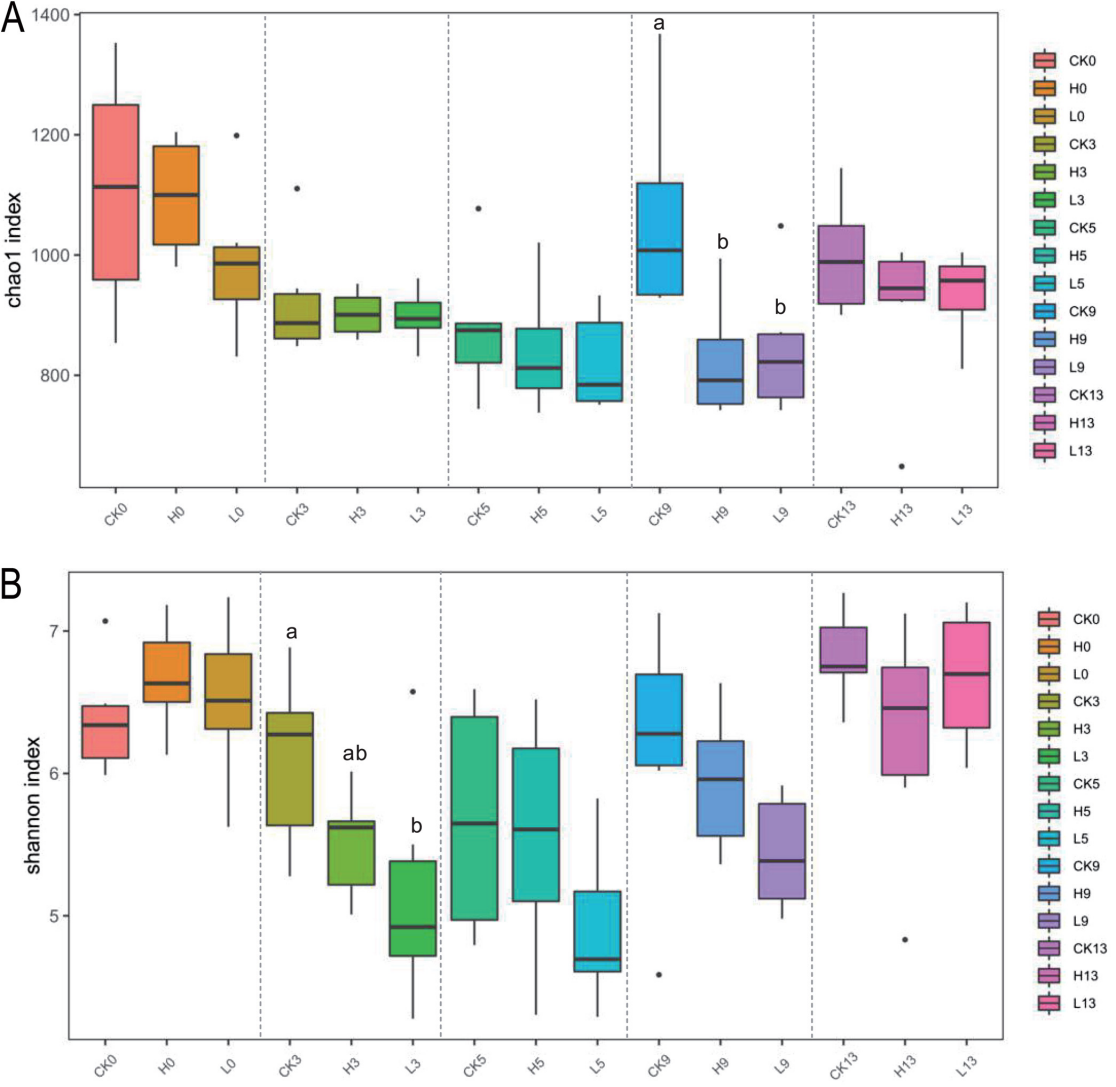

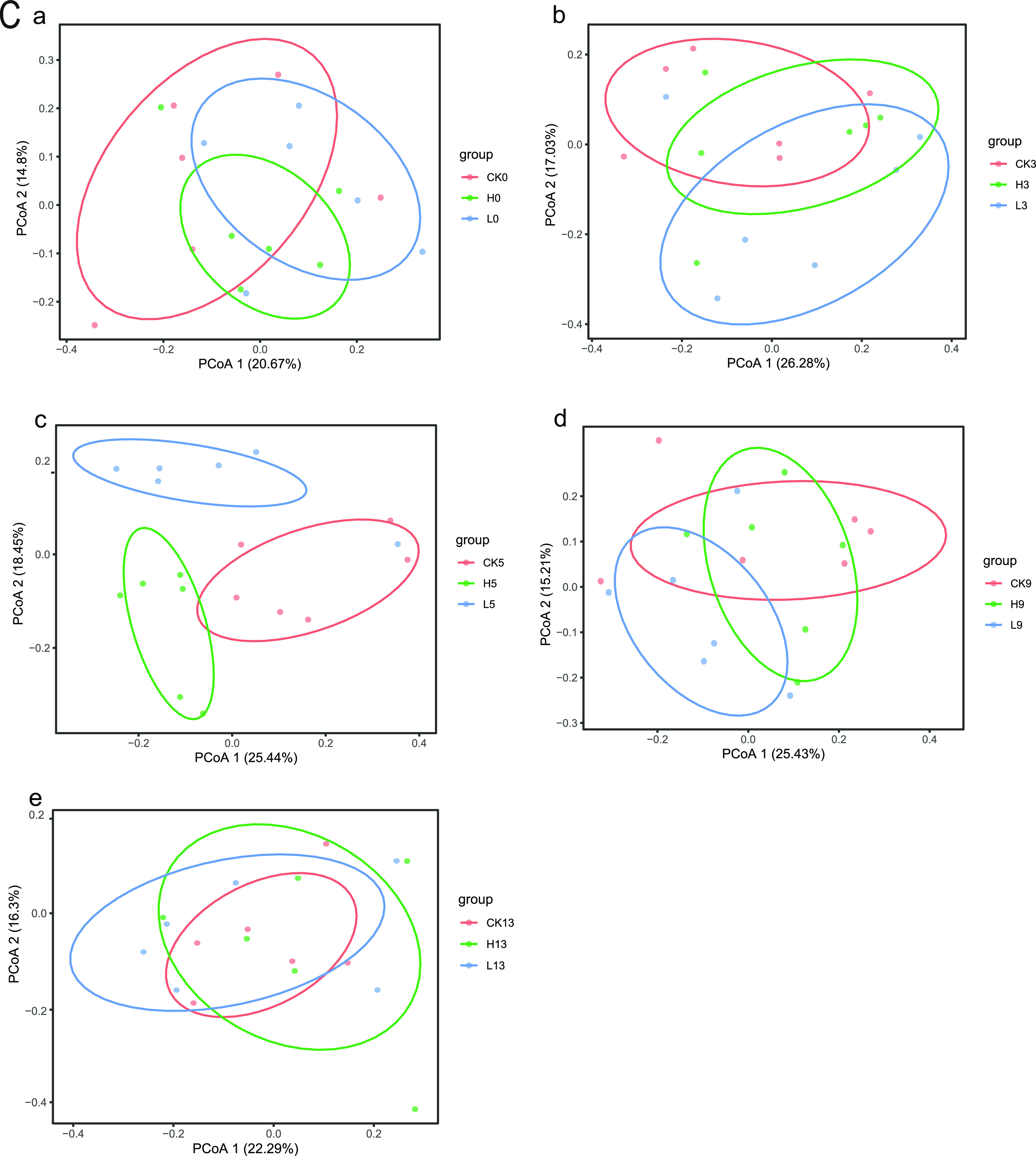
Changes in the diversity of the pig manure bacterial community after enrofloxacin treatment. (A, B) The α-diversity (Chao1 and Shannon indices) of the manure bacterial community. (C) The β-diversity of the manure bacterial community. Different letters above columns indicate significant differences (*P* < 0.05) among the three groups.

To determine whether enrofloxacin affected bacterial abundance, a 16S rRNA gene-targeting qPCR approach was applied. Enrofloxacin did not significantly affect the absolute abundance of total bacteria in pig manure (*P* > 0.05) (Fig. S1). The effect of enrofloxacin on bacterial community diversity was then explored (Fig. 1). The Chao1 and Shannon indices were used to quantify the bacterial α-diversity. In both the high concentration enrofloxacin (H) and low concentration enrofloxacin (L) groups, the Chao1 and Shannon index values showed a trend of first decreasing and then increasing (Fig. 1A and B). On the ninth day, the Chao1 index values of group H and group L were significantly lower than that of group CK (*P* < 0.05). In addition, on the third day, we found that the Shannon index of the H and L groups was significantly lower than that of the CK group.

Enrofloxacin treatment had a significant effect on the manure bacterial community β-diversity (*P* < 0.05). Principal coordinate analysis (PCoA) was used to quantify the bacterial community β-diversity. This β-diversity was based on binary Jaccard to reflect the degree of difference in abundance between samples. On day 0, groups CK, H, and L clustered together. With the use of enrofloxacin, the H and L groups gradually separated from the CK group on the third day. On the fifth day, the three groups were completely separated, but on the ninth day, the three groups gradually moved closer and finally regrouped on the thirteenth day (Fig. 1C). Treatment with different concentrations of enrofloxacin resulted in significant differences in the β-diversity of the bacterial communities (*P* < 0.05). Overall, the results indicated that exposure to enrofloxacin can reduce the manure bacterial community α-diversity and change the β-diversity, but the manure bacterial community diversity quickly recovered with enrofloxacin withdrawal.

### Influence of antibiotic stress on the pig manure bacterial community structure.

**FIG 2:**
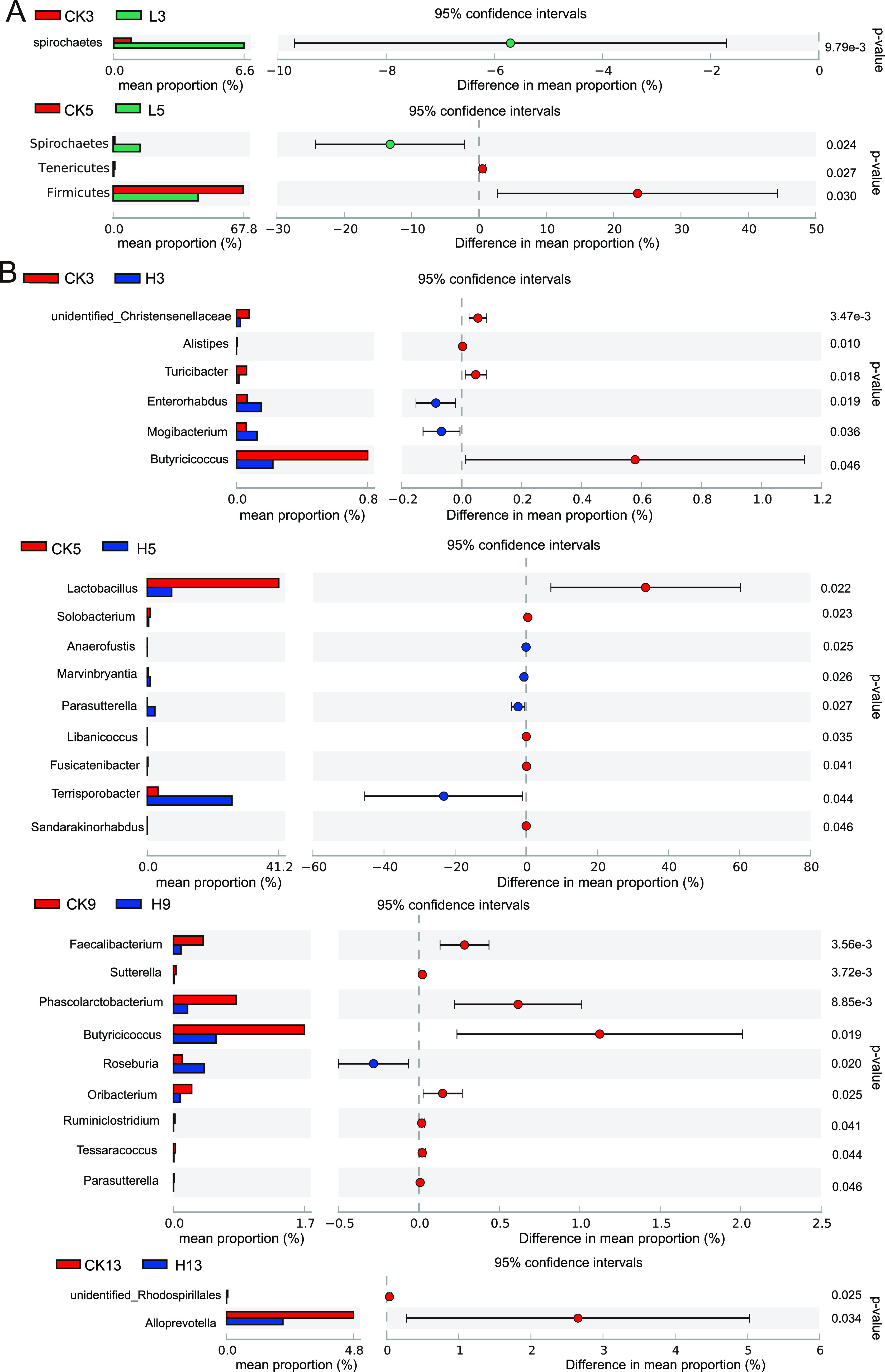

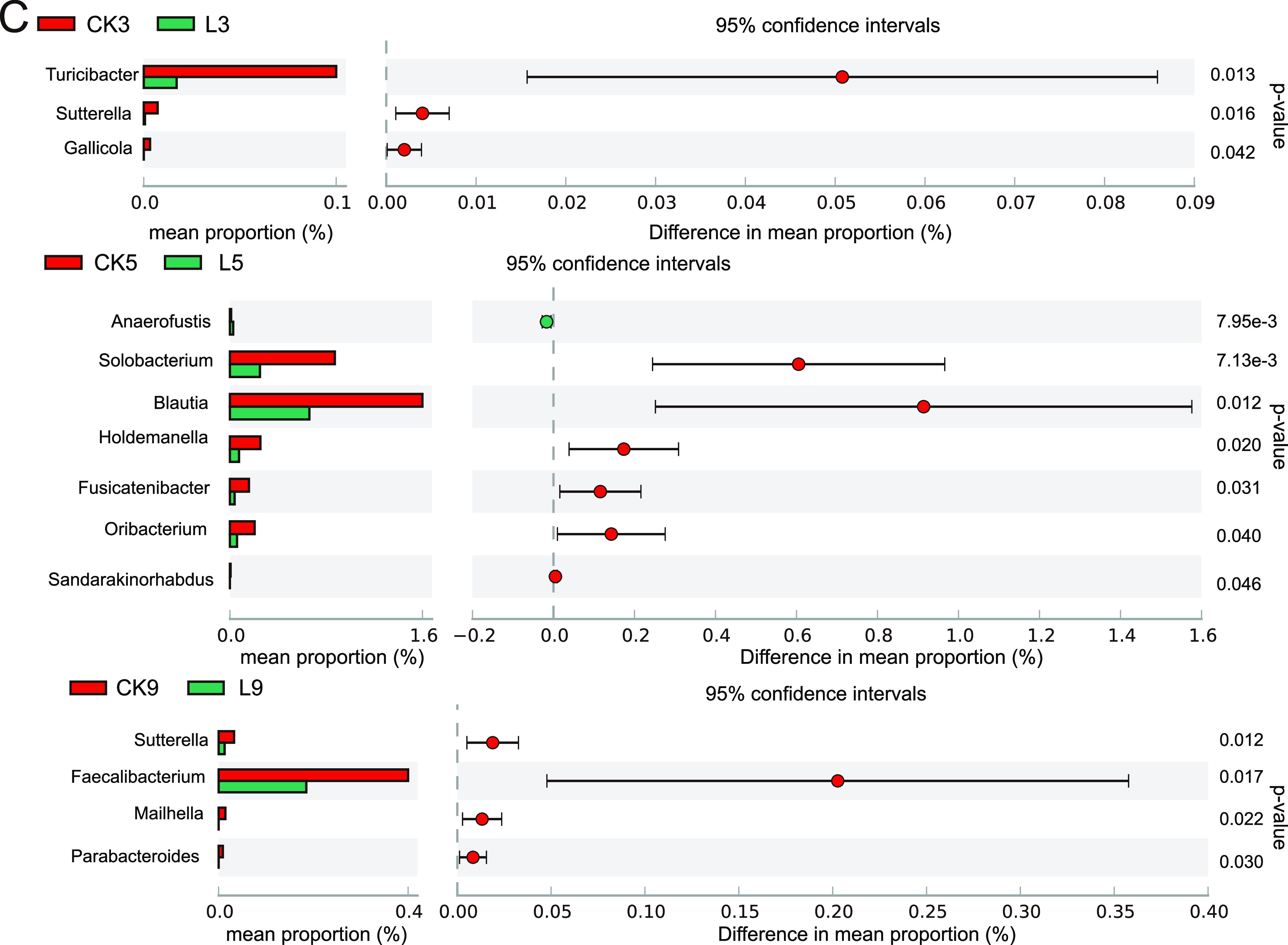
Changes in the structure of the pig manure bacterial community after enrofloxacin treatment. (A) The difference between the L group and the CK group at the phylum level. (B) The difference between group H and group CK at the genus level. (C) The difference between group L and group CK at the genus level.

To study whether the use of enrofloxacin affects the porcine manure bacterial structure, the bacterial community structure was determined by analyzing the 16S rRNA of bacteria. The dominant phyla mainly included Firmicutes, Bacteroidetes, Spirochaetes, and Proteobacteria (Fig. S2A). In addition, Firmicutes and Bacteroidetes were the main phyla and accounted for more than 75% of the bacterial community. The differences between group H and group L relative to group CK were determined (Fig. 2A). Among the three groups (CK, H, and L), we found that there was one differentially abundant bacterial phylum on the third day and three differentially abundant bacterial phyla on the fifth day, and the differentially abundant phyla disappeared on the ninth day. On the third day, the relative abundance of Spirochaetes in group L was significantly higher than that in group CK (*P* < 0.05). On the fifth day, the relative abundance of Firmicutes and Tenericutes in group L was reduced significantly compared with that in group CK (*P* < 0.05). In addition, on the fifth day, the relative abundance of Spirochaetes in group L was still significantly lower than that in group CK (*P* < 0.05). The difference in bacterial abundance disappeared on the ninth day. Until the thirteenth day, there was no difference in bacteria at the phylum level among the three groups (CK, H, and L).

The dominant bacterial genera in the pig manure were *Lactobacillus*, *Terrisporobacter,* and Streptococcus (Fig. S2B). Enrofloxacin treatment resulted in a significant change in bacterial abundance at the genus level. Differences were found between group H and group L relative to group CK. Similar to the results at the phylum level, with the progression of time, the number of differentially abundant bacterial genera showed a trend of first increasing and then decreasing (Fig. 2B and C). Compared with the CK group, there were 7 differentially abundant bacterial genera in group H, 5 of which showed significantly reduced abundances. On the fifth day, there were 9 differentially abundant bacterial genera, 5 of which showed significantly reduced abundances. On the ninth day, there were still 9 differentially abundant bacterial taxa, 5 of which showed significantly reduced abundances. On the thirteenth day, there were only two differentially abundant bacterial genera, both of which showed significantly reduced abundances, and these were different from the previously detected bacteria (Fig. 2B). Compared with the CK group, there were 3 differentially abundant bacterial genera in group H, 3 of which showed significantly reduced abundances. On the fifth day, there were 6 differentially abundant bacterial genera, 5 of which showed significantly reduced abundances. On the ninth day, there were 4 differentially abundant bacterial genera, all 4 of which showed significantly reduced abundances. On the thirteenth day, the differentially abundant bacterial genera disappeared completely (Fig. 2C). The results clearly showed that the changes caused by using enrofloxacin mainly resulted in reductions in bacterial abundance. Moreover, most of the differentially abundant bacteria (19/29) belonged to Firmicutes (Table S1).

### Effects of the physiochemical properties of pig manure on the bacterial community.

**FIG 3:**
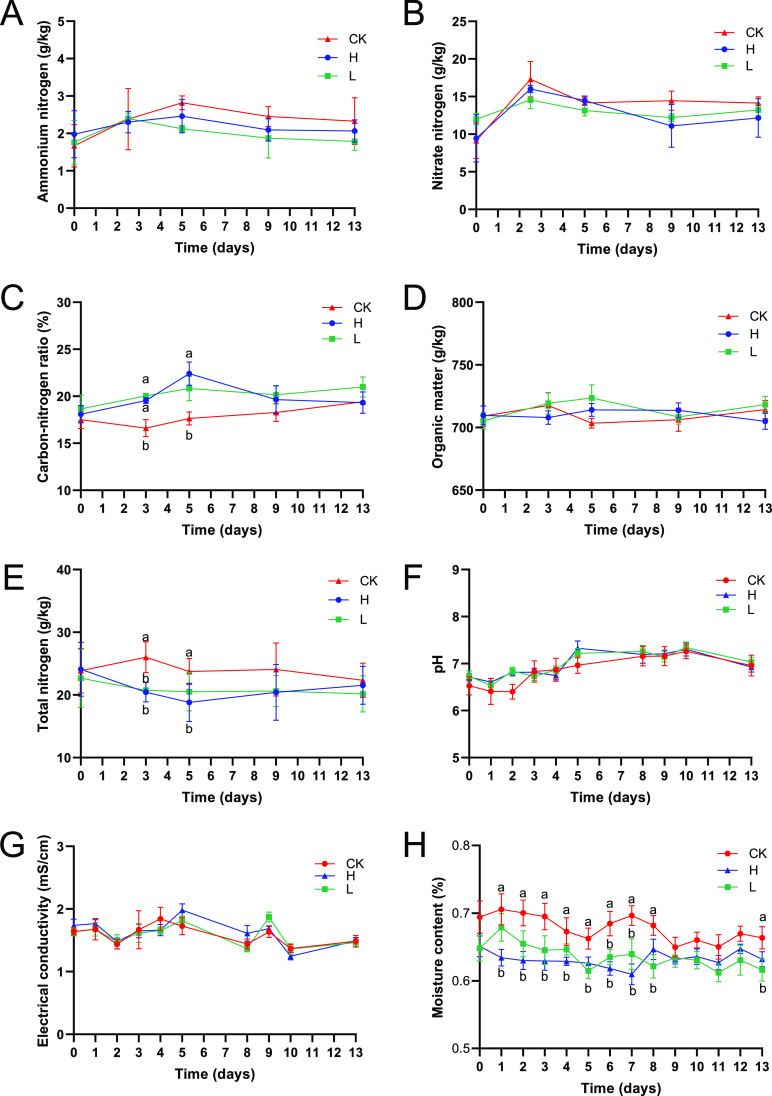
Changes in the main physicochemical properties of pig manure during the experiment. (A) Ammonium nitrogen (NH_4_^+^-N). (B) Nitrate nitrogen (NO_3_-N). (C) Carbon-nitrogen ratio (C/N). (D) Organic matter (OM). (E) Total nitrogen (TN). (F) pH. (G) Electrical conductivity (EC). (H) moisture content (MC).

**FIG 4:**
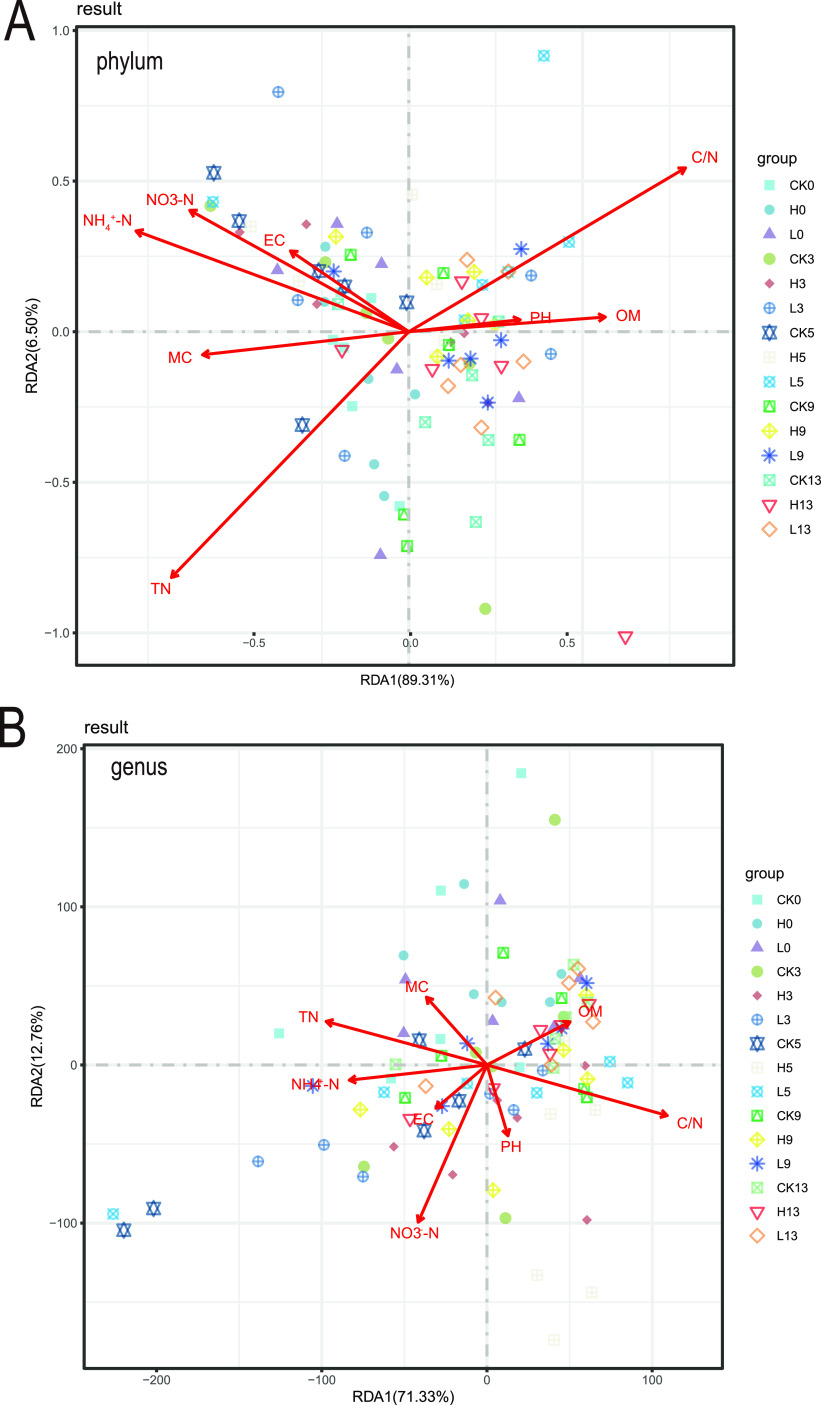
Redundancy analysis (RDA) of physiochemical properties and bacterial communities. (A) RDA of the manure bacterial community and physiochemical properties at the phylum level. (B) RDA of the manure bacterial community and physiochemical properties at the genus level.

The effects of enrofloxacin on physiochemical properties were examined. This study measured eight major physicochemical properties: electrical conductivity (EC), ammonium nitrogen (NH_4_^+^-N), nitrate-nitrogen (NO_3_-N), total nitrogen (TN), organic matter (OM), carbon-nitrogen ratio (C/N), moisture content (MC) and pH. The results in Fig. 3 show that the EC, NH_4_^+^-N, NO_3_-N, TC, and pH did not change significantly, but the MC, TN, and C/N changed significantly. On the third and fifth days, the TN of group H was significantly lower than that of group CK, and the difference disappeared on the ninth day. We found that the difference in C/N could be attributed to the difference in TN. Physiochemical properties are generally considered to be the driving force affecting the bacterial community structure. Therefore, we performed a redundancy analysis (RDA) between physiochemical properties and bacterial communities. At the phylum level, the results of the RDA of the bacterial community and physiochemical properties showed that the physiochemical properties explained 95.81% of the changes in the manure bacterial community (Fig. 4A). Similarly, at the genus level, the results of the RDA of the bacterial community and physiochemical properties showed that the physiochemical properties explained 84.09% of the changes in the manure bacterial community (Fig. 4B). NO3-N, NH4^+^-N, TN, and C/N were the main driving factors affecting the changes in the manure bacterial community, and these physicochemical properties are all related to nitrogen. Combined with the significant changes observed in TN and C/N, these results indicate that nitrogen metabolism has the greatest impact on the structure of the manure bacterial community.

### Influence of antibiotic stress on the functional genes of the pig manure bacterial community.

**FIG 5:**
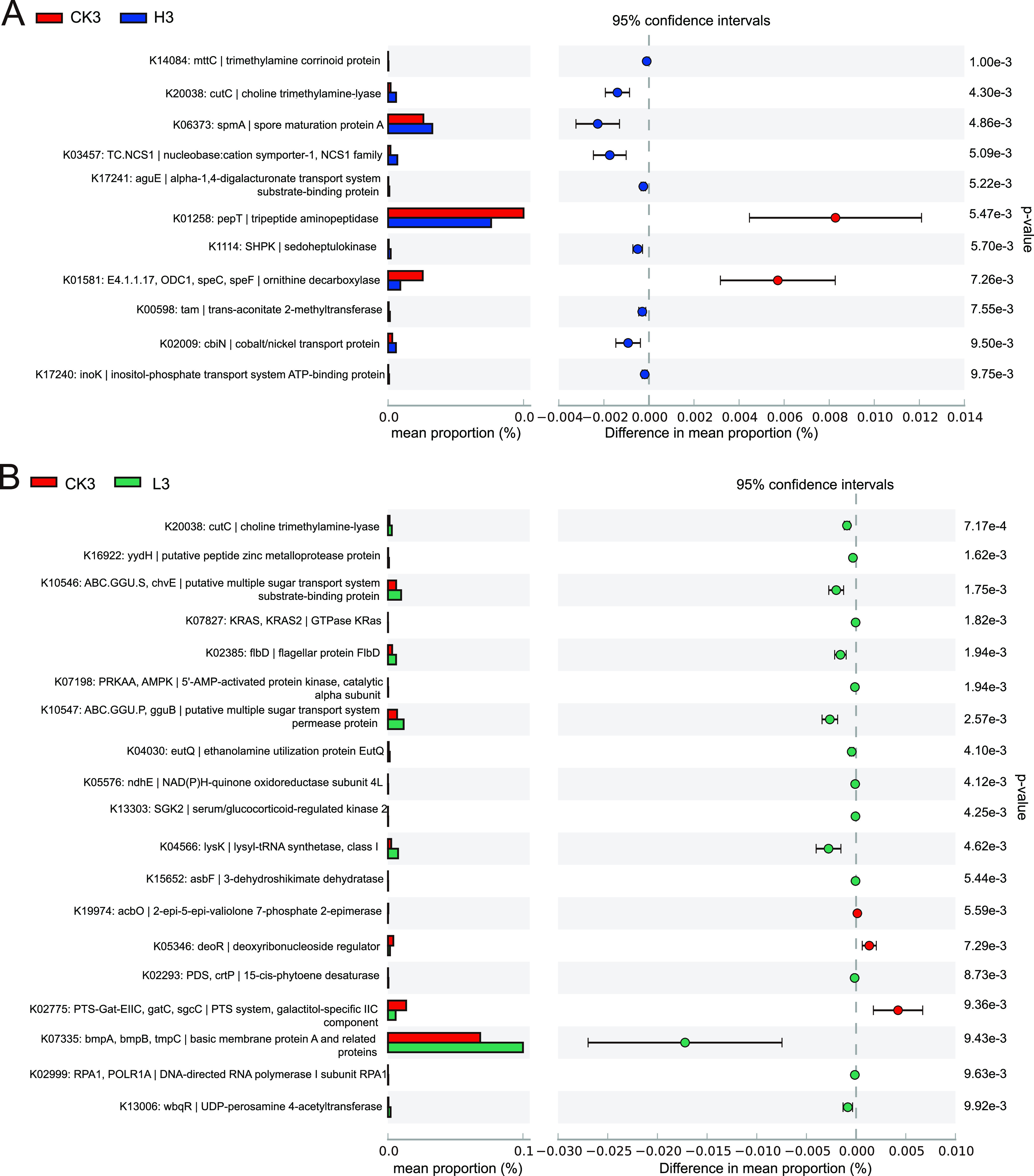

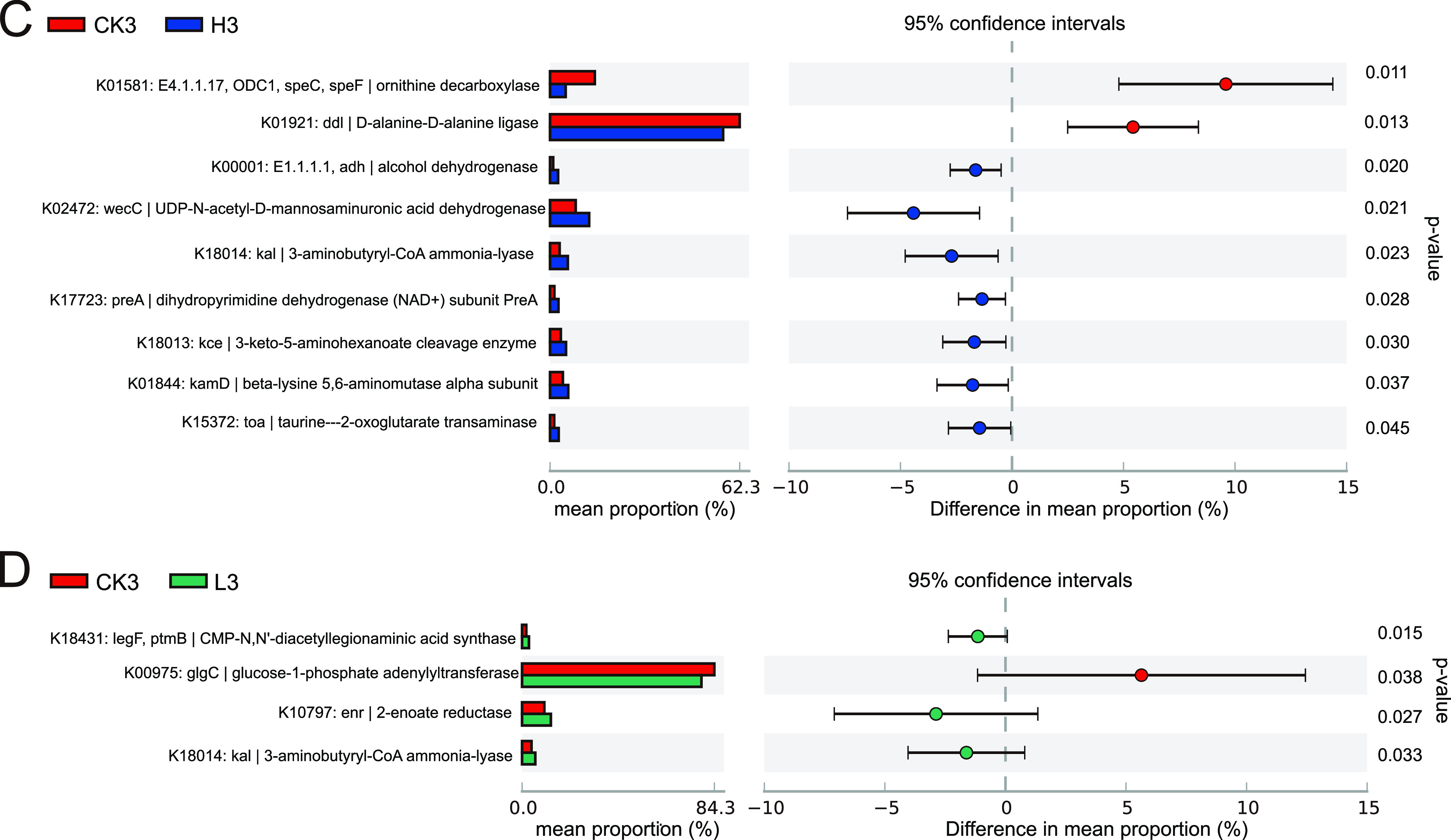
Changes in the abundance of functional genes of the pig manure bacterial community after enrofloxacin treatment. (A) On the third day, the KOs were different between group CK and group H (*P* < 0.01). (B) On the third day, the KOs were different between group CK and group L (*P* < 0.01). (C) On the third day, nitrogen metabolism-related KOs were different between group CK and group H (*P* < 0.05). (D) On the third day, nitrogen metabolism-related KOs were different between the group CK and the L group (*P* < 0.05).

Metagenomic sequencing was used to analyze the changes in porcine manure bacterial community function. A total of 1,868,626 open reading frames (ORFs) were obtained after gene prediction and deredundancy analysis of sequencing data. Through functional database annotation of nonredundant gene sets, 1,091,292 (58.40%) ORFs were aligned to the KEGG database. The differentially abundant functional genes in group H and group L relative to group CK were identified (*P* < 0.01). On the third day, compared with the CK group, there were significant differences in the 11 KEGG Orthology (KOs) in group H, 6 of which were attributed to the metabolism pathway, while 3 belonged to the environmental information processing pathway (Fig. 5A, Table S2). Moreover, most of the differentially abundant KOs (9/11) were significantly enriched (*P* < 0.01), and only 2/11 KOs were significantly depleted (*P* < 0.01). Additionally, 4/6 KOs belonging to the metabolism pathway showed significant enrichment. Similar to group H, compared with the CK group, there were significant differences in the 19 KOs in the L group, 19 of which were attributed to the metabolism pathway, while 5belonged to the environmental information processing pathway (Fig. 5B). Most of the KOs (16/19) were significantly enriched, and only 3/19 KOs were significantly depleted. The 7/9 KOs belonging to the metabolic pathway showed significant enrichment. On the thirteenth day, all the above differences disappeared.

Enrofloxacin stress resulted in a significant decrease in the TN content in pig manure (Fig. 3E). Therefore, we performed an analysis of differentially abundant KOs involved in nitrogen metabolism. Compared with the CK group, 7/9 nitrogen metabolism-related differentially abundant KOs in group H were significantly enriched (Fig. 5C), and 3/4 nitrogen metabolism-related differentially abundant KOs in group L were significantly enriched (Fig. 5D). Therefore, the increase in nitrogen metabolism in the pig manure bacterial community was consistent with the decrease in TN content in pig manure.

## DISCUSSION

Previous studies have shown that antibiotics destroy the structure of the intestinal bacterial communities of livestock and poultry (37) and may lead to the development of antibiotic resistance (38). Because antibiotic treatment is still a common and effective practice in livestock and poultry breeding (39), it is very important to determine the influence of antibiotics on the intestinal bacterial community at therapeutic concentrations. This study referred to the therapeutic concentration of enrofloxacin in the Veterinary Pharmacopoeia of the People's Republic of China to set the injection concentration. Enrofloxacin did not cause significant changes in total bacterial abundance at therapeutic concentrations, but it caused great changes in bacterial diversity and bacterial community structure. Enrofloxacin treatment led to a decrease in bacterial diversity, which is consistent with previous studies of other antibiotics (11, 40). At present, it is generally accepted that the diversity of bacteria is beneficial for intestinal bacterial stabilization (41). One important way in which diversity can confer resilience is through a wide repertoire of responses to stress, which is referred to as the insurance hypothesis (42). A decrease in bacterial diversity indicates that the stability of the bacterial community is reduced in response to external stimuli. Under enrofloxacin stress, the structure of the manure bacterial community changed. Higher concentrations of enrofloxacin resulted in more significant changes in abundance at the genus level than lower concentrations of enrofloxacin. Most differentially abundant bacteria at the genus level belonged to Firmicutes, among which *Terrisporobacter* harbored the quinolone antibiotic resistance gene (ARG) *tnp*A (43), which may be one of the reasons for its increased abundance. After enrofloxacin treatment, most of the differentially abundant bacterial genera showed a decrease in abundance. This might be because these bacterial genera were not hosts of quinolone resistance genes and could not confer resistance to enrofloxacin. Therefore, bacteria with increased abundance after antibiotic treatment may be worthy of attention (44).

Most antibiotic treatments cause changes in the intestinal bacterial community, but the persistence of these changes differs. Amoxicillin treatment increased Proteobacteria abundance and decreased the Firmicutes abundance. These differences disappeared 3 days after stopping treatment (13). Similarly, 14 days after stopping carbadox treatment, the pig manure bacterial community was restored (45). In addition, some people believe that antibiotics have only a long-term effect on the intestinal bacterial community. After 97 days of treatment, the relative abundance of Proteobacteria in the gut of female pigs increased 1.8-fold (12). Whether these antibiotic-induced differences in the microbiota and host physiology revert or remain after treatment may depend on whether individuals receive a single dose of antibiotics or whether they are chronically exposed (41). Therefore, in this study, we analyzed whether enrofloxacin has persistent effects on the porcine manure bacterial community. We found that, although enrofloxacin treatment caused changes in the structure and diversity of the manure bacterial community, the phylum-level bacterial community structure and diversity returned to the level of the control group on the thirteenth day. In summary, studies on the structure and diversity of the manure bacterial community have shown that enrofloxacin may not have a lasting impact on the porcine manure bacterial community.

When studying the effect of antibiotics on the intestinal bacterial community, the changes in the function of the bacterial community are often overlooked. Intestinal bacteria can ferment indigestible food ingredients into absorbable metabolites, synthesize essential vitamins, and remove toxic compounds (46, 47). Moreover, the intestinal bacterial community acts as an endocrine organ by producing bioactive metabolites that can directly or indirectly affect the physiology of the host (35). However, previous studies have shown that antibiotics can disrupt the function of the intestinal bacterial community (11, 45). For example, studies on piglets have shown changes in the function of the phosphotransferase system in the intestinal bacterial community after antibiotic intervention in early life (16). Therefore, we studied whether enrofloxacin destroys the function of the intestinal bacterial community and after the effects of discontinuation of enrofloxacin treatment. In this study, metagenomic sequencing was used to analyze the changes in porcine manure bacterial function. The KEGG database was used for gene functional annotation. During the medication period, most of the differentially abundant KOs under enrofloxacin treatment were significantly upregulated, and they were mainly concentrated in the metabolism pathway. Maintaining high energy metabolism during antibiotic therapy is beneficial for antibiotic resistance and for restoring growth after antibiotic therapy (48). In this study, enrofloxacin treatment caused significant changes in TN, C/N, and MC in pig manure, among which TN was significantly reduced. Physiochemical properties are considered to be highly correlated with changes in the bacterial community structure (49). RDA was performed between physiochemical properties and bacterial communities. The physicochemical properties were correlated with changes in pig manure bacterial communities at the phylum and genus levels. Among them, N-related physiochemical properties were the most closely related to the changes in the bacterial communities. Antibiotics can enhance nutrient absorption and utilization, which is closely related to animal health. Many factors are involved in this phenomenon, including nutrient transport and intestinal morphology. This alteration is mainly mediated by the interaction between the host and the bacterial community in the intestine (50, 51). In this study, the significant reduction in TN content in pig manure after treatment with enrofloxacin might be related to the significant increase in the abundance of functional genes related to nitrogen metabolism pathways. On the thirteenth day, these functional genetic differences completely disappeared, which is consistent with the change in TN content in pig manure. The results of this study indicate that the use of enrofloxacin as a therapeutic drug may not have a lasting effect on the manure bacterial community of pigs.

Both pork and groundwater can have high concentrations of enrofloxacin residues, so the human intestinal bacterial community is also at risk of exposure to enrofloxacin (24, 52, 53). This study revealed that exposure to enrofloxacin can cause changes in the structure and function of the intestinal bacterial community. This effect of enrofloxacin on the intestinal bacterial community may not be long-lasting. However, previous studies have shown that enrofloxacin residues in edible animal products may have adverse effects on humans, such as inducing allergic allergies, reducing sperm count, and diminishing sperm motility (54, 55). Therefore, although enrofloxacin exposure did not cause permanent changes in the manure bacterial community, attention should be given to its effects on other organs. In addition, the pig manure bacterial community was used in this study to study the pig intestinal bacterial community, which has certain reliability, but due to the difference between the two, the use of intestinal contents should be directly considered for future research.

Overall, this study demonstrated that enrofloxacin treatment caused changes in the structure and function of the pig manure bacterial community. However, the manure bacterial community structure and function may recover rapidly after the discontinuation of enrofloxacin treatment. During the medication period, most of the differentially abundant functional genes of the manure bacterial community were significantly upregulated, especially genes related to metabolic pathways, for adaptation to the antibiotic environment. During this process, nitrogen metabolism in the manure bacterial community was significantly upregulated, which is consistent with the significant decrease in the TN content of pig manure.

## MATERIALS AND METHODS

### Animal experiments and sample collection.

Fifteen-week-old Duroc-Landrace-Yorkshire barrows (weight 57.11 ± 5.88 kg, *n* = 18) selected from populations with similar genetic backgrounds were used as experimental subjects. All groups were also provided identical diets, which met their nutrient requirements as previously described (56). These pigs were randomly divided into three groups (CK, H, and L). Pigs in group H were injected with 5 mg/kg body weight enrofloxacin, pigs in group L were injected with 2.5 mg/kg body weight enrofloxacin, and pigs in group CK were injected with an equal volume of normal saline. The injected concentration of enrofloxacin was based on the “Veterinary Pharmacopoeia of the People’s Republic of China (2015)”. The maximum concentration specified in the Pharmacopoeia was 2.5 mg/kg body weight. These pigs were prefed for 7 days to allow them to adapt to the experimental environment. The experimental period was 13 days, of which days 1 to 3 were the medication period and days 4 to 13 were the drug withdrawal period. Fresh pig manure was collected every day from the day before the start of the experiment to the thirteenth day of the experiment. These samples were packed into a 50 mL sterile centrifuge tube and stored at −80°C. All animal experiments were performed in accordance with the Institutional Animal Care, National (GB 13078–2001 and GB/T 17237–1998) and Agricultural Standards (NY 5148-2002-NY 100 5151-2002) of the People’s Republic of China. This study was approved by the Ethics Committee of South China Agricultural University, and the methods were carried out in accordance with the regulations and guidelines established by this committee.

### DNA extraction and bacterial 16S rRNA gene sequencing.

A total of 90 pig manure samples were collected on days 0, 3, 5, 9, and 13 for 16S rRNA sequencing. Total microbial DNA was extracted using the Omega E.Z.N.A. TM Soil DNA kit (Omega, USA) according to the manufacturer's instructions. The hypervariable regions (V3 to 4) of the bacterial 16S rRNA gene were amplified using the barcoded bacterium-specific primers 341 F (5′-CCTAYGGGRBGCASCAG-3′) and 806 R (5′-GGACTACHVGGGTWTCTAAT-3′). After the reaction, mixed PCR products were purified with a GeneJET Gel Extraction kit (Thermo Scientific). Sequencing libraries were then generated using an Ion Plus Fragment Library Kit 48 (Thermo Scientific) following the manufacturer's recommendations. Library quality was assessed on a Qubit 2.0 Fluorometer (Thermo Scientific). Finally, the library was sequenced on a NovaSeq6000 platform (Novogene, China). The clean data were clustered into operational taxonomic units (OTUs) for species classification under 0.97 similarity, and the bacterial α-diversity (Shannon and Chao 1 indices) was calculated by QIIME 1.9.1. The β-diversities of the treatments were compared by principal coordinate analysis (PCoA) based on Bray-Curtis distances.

### Bacterial metagenomic sequencing.

A total of 54 samples were collected on days 0, 3, and 13. For day 0 samples, six replicates per group were pooled into one sample, and the day 3 and 13 samples were pooled in pairs with six replicates per group into three samples. Total microbial DNA was extracted using an Omega E.Z.N.A. TM Soil DNA kit (Omega, USA). Metagenomic sequencing libraries were generated with an insert size of 350 bp (bp) of DNA following the manufacturer’s instructions (Illumina, San Diego, CA, United States). The libraries for metagenomic analysis were sequenced on an Illumina HiSeq 2500 platform by the Illumina HiSeq-PE150 method (Novogene, China). Preprocessing of the raw data obtained from the Illumina HiSeq sequencing platform using Readfq was conducted to acquire clean data for subsequent analysis. SOAP *de novo* assembly software was used for assembly analysis. MetaGeneMark was used for ORF prediction. DIAMOND software (V0.9.9) was adopted to blast unigenes against functional databases with the parameter setting of blastp, “-e 1e−5”. The functional database contained the KEGG, eggNOG, and CAZy database information.

### Quantification of bacteria.

The abundance of bacteria was measured by qPCR. The 16S rRNA-specific primers for qPCR included the forward primer CGGCAACGAGCGCAACCC and the reverse primer CCATTGTAGCACGTGTGTAGCC. The construction of recombinant plasmids carrying ARGs and the calculation method for absolute abundance were as described in a previous study (57). All qPCRs were run on a BioRad CFX96 PCR System (BioRad, USA). The total qPCR system volume of 25 mL comprised 1 mL of DNA, 12.5 μL of SYBR Premix Ex Taq (TaKaRa), 10.5 μL of ddH2O, 0.5 μL of each primer (10 μM), and 1 μL of sample DNA. The thermal cycle was as follows: (i) predenaturation at 95°C for 4 min and (ii) 40 cycles of denaturation at 95°C for 15 s, annealing at 55°C for 30 s, and extension at 75°C for 30 s. Three technical replicates for each sample were performed. The absolute abundances of bacteria were expressed as the logarithm of copies/g of dry matter (DM).

### Measurement of physicochemical properties.

Manure pH was measured in a suspension of 1:5 manure:water with a pH meter. Manure conductivity was measured in a suspension that contained 2 g of manure and 20 mL of water with a benchtop conductivity meter (INESA Scientific Instrument Co., Ltd., China). The manure moisture content was measured after drying in a drying oven (ZH Instrument, China) at 105°C. The measurements of the manure OM, TN, NO_3_-N, and NH_4_^+^-N were performed according to previously reported methods (58).

### Data analysis.

SPSS 20.0 (IBM, USA) was used to perform an analysis of variance (ANOVA) and Tukey's test to analyze physicochemical properties, KOs, and bacterial community structure data in different groups. Differences in bacteria between groups were assessed by Welch's *t* test. Differences were considered statistically significant at *P* < 0.05.

### Data availability.

The raw sequence data reported in this paper have been deposited in the Genome Sequence Archive (59), Beijing Institute of Genomics (BIG), BIG Data Center (60), and Chinese Academy of Sciences under accession number CRA006297.
