# Supplementary material for: Recovery of the Structure and Function of the Pig Manure Bacterial Community after Enrofloxacin Exposure
Source: Microbiol Spectr. 2022 May 23;10(3):e02004-21. doi: 10.1128/spectrum.02004-21 (PMC9241743; doi:10.1128/spectrum.02004-21)
Supplement: SUPPLEMENTAL FILE 1 — Supplemental material. Download spectrum.02004-21-s001.pdf, PDF file, 0.3 MB [file spectrum.02004-21-s001.pdf]

Table S1. The phyla corresponding to bacteria with genus-level differences.

**genus**	**phylum**	**genus**	**phylum**
unidentified_Christensenellaceae	Firmicutes	Fusicatenibacter	Firmicutes
Turicibacter	Firmicutes	Terrisporobacter	Firmicutes
Mogibacterium	Firmicutes	Blautia	Firmicutes
Butyricicoccus	Firmicutes	Holdemanella	Firmicutes
Gallicola	Firmicutes	Oribacterium	Firmicutes
Lactobacillus	Firmicutes	Faecalibacterium	Firmicutes
Solobacterium	Firmicutes	Phascolarctobacterium	Firmicutes
Anaerofustis	Firmicutes	Roseburia	Firmicutes
Marvinbryantia	Firmicutes	Ruminiclostridium	Firmicutes
Parabacteroides	Firmicutes	Tessaracoccus	Actinobacteria
Parasutterella	Proteobacteria	Enterorhabdus	Actinobacteria
Mailhella	Proteobacteria	Libanicoccus	Actinobacteria
unidentified_Rhodospirillales	Proteobacteria	Alloprevotella	Bacteroidetes
Sutterella	Proteobacteria	Alistipes	Bacteroidetes
Sandarakinorhabdus	Proteobacteria		

Table S2. The differentially abundant KOs corresponding to KEGG pathways.

Time	KO_ID	Definition	KEGG pathway
3rd day	K17241	aguE | alpha-1,4-digalacturonate transport system substrate-binding protein	Environmental Information Processing
3rd day	K01581	E4.1.1.17, ODC1, speC, speF | ornithine decarboxylase	Metabolism
3rd day	K03457	TC.NCS1 | nucleobase:cation symporter-1, NCS1 family	Cellular Processes
3rd day	K02009	cbiN | cobalt/nickel transport protein	Environmental Information Processing
3rd day	K20038	cutC | choline trimethylamine-lyase	metabolism
3rd day	K17240	inoK | inositol-phosphate transport system ATP-binding protein	Environmental Information Processing
3rd day	K14084	mttC | trimethylamine corrinoid protein	Metabolism
3rd day	K01258	pepT | tripeptide aminopeptidase	Metabolism
3rd day	K11214	SHPK | sedoheptulokinase	Metabolism
3rd day	K06373	spmA | spore maturation protein A	Cellular processes
3rd day	K00598	tam | trans-aconitate 2-methyltransferase	Metabolism
3rd day	K10547	ABC.GGU.P, gguB | putative multiple sugar transport system permease protein	Environmental Information Processing
3rd day	K10546	ABC.GGU.S, chvE | putative multiple sugar transport system substrate-binding protein	Environmental Information Processing
3rd day	K07335	bmpA, bmpB, tmpC | basic membrane protein A and related proteins	Environmental Information Processing
3rd day	K07827	KRAS, KRAS2 | GTPase KRas	Environmental Information Processing
3rd day	K04566	lysK | lysyl-tRNA synthetase, class I	Genetic Information Processing
3rd day	K02293	PDS, crtP | 15-cis-phytoene desaturase	Metabolism
3rd day	K07198	PRKAA, AMPK | 5'-AMP-activated protein kinase, catalytic alpha subunit	Environmental Information Processing
3rd day	K02775	PTS-Gat-EIIC, gatC, sgcC | PTS system, galactitol-specific IIC component	Metabolism

3rd day	K02999	RPA1, POLR1A | DNA-directed RNA polymerase I subunit RPA1	Genetic Information Processing
3rd day	K19974	acbO | 2-epi-5-epi-valiolone 7-phosphate 2-epimerase	Metabolism
3rd day	K15652	asbF | 3-dehydroshikimate dehydratase	Metabolism
3rd day	K20038	cutC | choline trimethylamine-lyase	metabolism
3rd day	K05346	deoR | deoxyribonucleoside regulator	genetic information processing
3rd day	K04030	eutQ | ethanolamine utilization protein EutQ	metabolism
3rd day	K02385	flbD | flagellar protein FlbD	cellular processes
3rd day	K05576	ndhE | NAD(P)H-quinone oxidoreductase subunit 4L	Metabolism
3rd day	K13303	SGK2 | serum/glucocorticoid-regulated kinase 2	Environmental Information Processing
3rd day	K13006	wbqR | UDP-perosamine 4-acetyltransferase	metabolism
3rd day	K16922	yydH | putative peptide zinc metalloprotease protein	metabolism

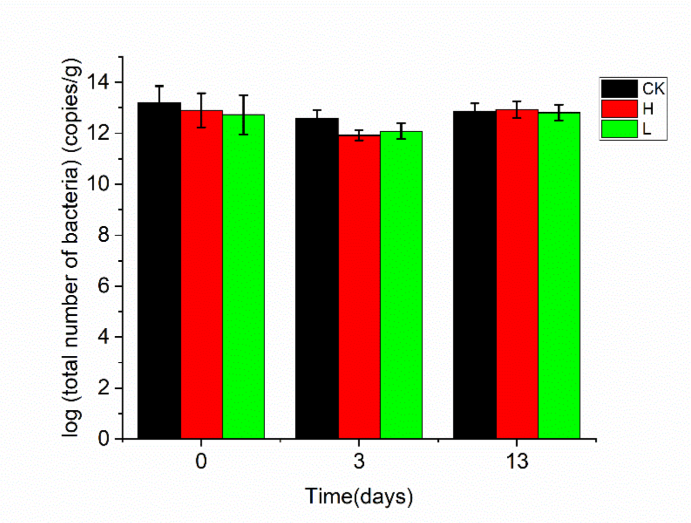

Fig S1 Changes in the total number of pig manure bacteria after enrofloxacin treatment.

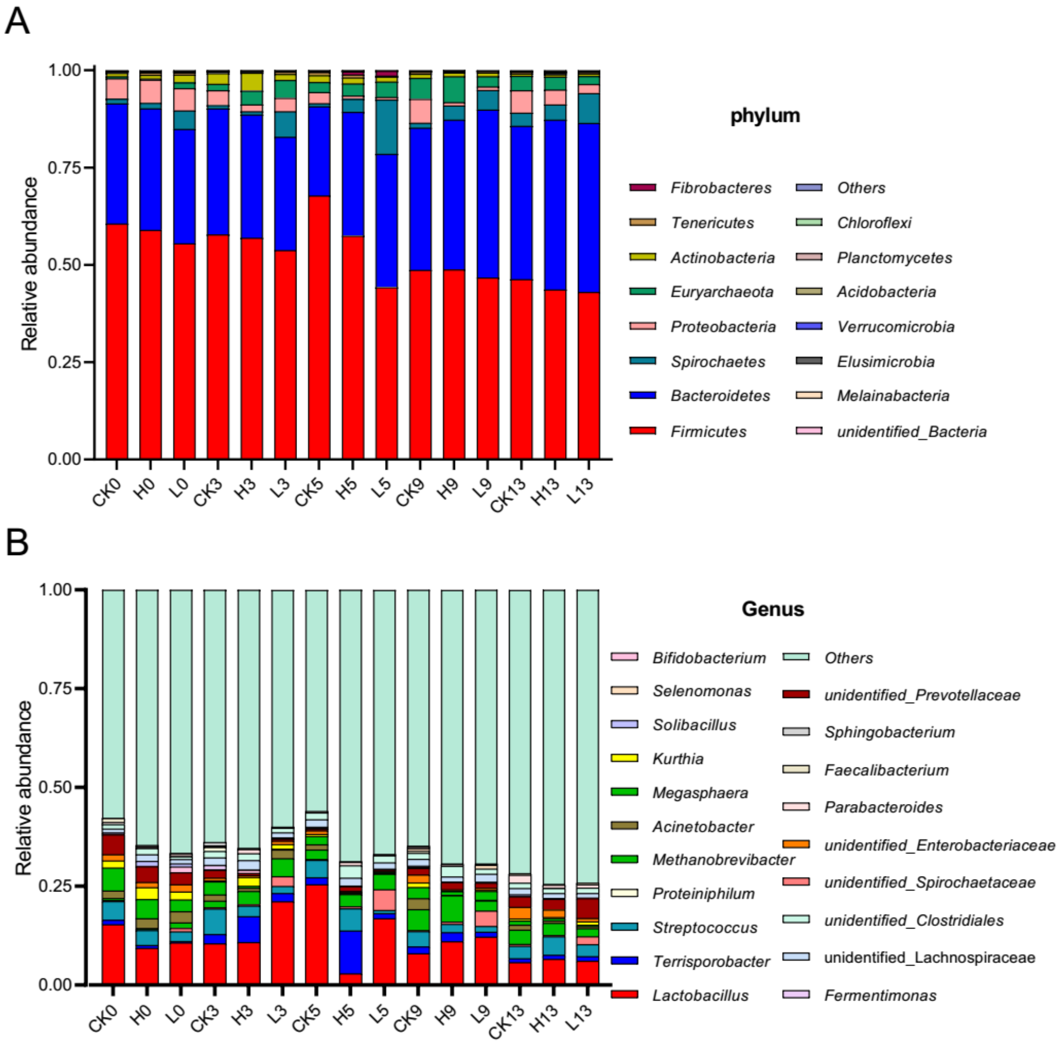

Fig S2 Pig manure bacterial community structure. (A) The relative abundance of bacteria at the phylum level. (B) The relative abundance of bacteria at the genus level.
